# Spiritual Leadership on Proactive Workplace Behavior: The Role of Organizational Identification and Psychological Safety

**DOI:** 10.3389/fpsyg.2019.01206

**Published:** 2019-05-22

**Authors:** Silu Chen, Wanxing Jiang, Guanglei Zhang, Fulei Chu

**Affiliations:** ^1^School of Economics and Business Administration, Central China Normal University, Wuhan, China; ^2^School of Business Administration, Shanghai Lixin University of Accounting and Finance, Shanghai, China; ^3^School of Management, Wuhan University of Technology, Wuhan, China; ^4^College of Business Administration, Capital University of Economics and Business, Beijing, China

**Keywords:** spiritual leadership, proactivity, organizational identification, psychological safety, intrinsic motivation, self-determination theory

## Abstract

This study investigates whether and why spiritual leadership may contribute to enhanced proactive employee workplace behavior. Based on self-determination theory (SDT), we examine the effects of two sequential mediators (i.e., organizational identification and psychological safety) on the relationship between spiritual leadership and proactive workplace behavior. Data collected from 188 subordinate-leader dyads in Chinese firms suggested that spiritual leadership has a significant positive effect on proactive workplace behavior. In addition, both organizational identification and psychological safety mediate the relationship between spiritual leadership and proactive workplace behavior. Furthermore, spiritual leadership positively influences organizational identification, and such identification nurtures psychological safety, which, in turn, fosters the proactive behavior of employees. It extends the existing impact of spiritual leadership to proactive workplace behavior and shed lights on the mediating mechanisms through which spiritual leadership exerts influences on proactive workplace behavior. Finally, it considers the important roles played by leaders in modern organizations.

## Introduction

Issues regarding workplace spirituality have received increasing attention from both theorists and practitioners (Mitroff and Denton, 1999; Giacalone and Jurkiewicz, 2003; Fry and Matherly, 2006). An increasing number of scholars are arguing that “spirituality” is necessary in organizations (Benefiel, 2005). As leaders play a major role in shaping employees’ attitudes and behaviors, it has become increasingly important for leaders to nurture a thriving and energized workforce that is ready to cope with various issues and tasks (Yang et al., 2017). Previous leadership theories have focused in varying degrees on one or more aspects of the physical, mental, or emotional elements of human interaction in organizations while neglecting the spiritual components (Fry, 2003). Spiritual leadership focuses on satisfying employees’ spiritual needs and values employees’ perceptions of meaningfulness at work, which can be defined as “comprising the values, attitudes, and behaviors that are necessary to intrinsically motivate one’s self and others so that they have a sense of spiritual survival through calling and membership” (Fry, 2003, p. 711). In other words, spiritual leadership differs from previous leadership theories in that it is a more spirit-centered and value-based approach to leadership (Fry, 2003; Yang et al., 2017). For example, although charismatic leadership and ideological leadership are vision-based leaderships, they often display some behavioral differences (Strange and Mumford, 2002). One the one hand, charismatic leaders stress the need for change, articulate a better future through change, evidence responsiveness to followers whose reactions are usually a source of meaning, and maintain a relatively close supportive relationship with followers (Mumford and Strange, 2013). On the other hand, when ideological leadership stresses values, standards, and the meaningfulness of these standards, followers will be of interest, not as entities unto themselves, but rather as their actions impinge on the values and standards being defined by the leader (Strange and Mumford, 2002). Compared with such leadership styles, spiritual leadership not only focuses on vision formation, but treats the spiritual domain as an integral component of leadership and uses spirituality as an important variable of an integrated leadership development model (Dent et al., 2005).

Recently, spiritual leadership in organizations has come to be considered as pertinent issue in management literature and organizational behavior. Existing research shows that spiritual leadership has a positive influence on individual and organizational-level outcomes, including organizational commitment and productivity (Fry et al., 2017), organizational citizenship behavior (Chen and Yang, 2012), pro-environmental behavior (Afsar et al., 2016), organizational transformation (Benefiel, 2005), and effective core organizational values (Ferguson and Milliman, 2008). However, despite the research on the impacts of spiritual leadership, it remains unclear how it lead to employees’ proactive behaviors. In fact, with an increasing demand for proactivity in organizations due to the rapid changes and uncertainty in the current business environment (Parker et al., 2006; Park and Jo, 2017), more scholars have begun to investigate the factors which could incite proactive workplace behaviors. For instance, Den Hartog and Belschak (2012) found that transformational leadership was positively related to employees’ proactive behavior. Erkutlu (2012) found that shared leadership within a work team was positively related to proactive team behavior. Accordingly, this research aims at investigating the linkage between spiritual leadership, i.e., “a means of spiritual awakening in the workplace” (Garcia-Zamor, 2003, p. 355) and proactive behavior. Moreover, responding to the call for further research on the mediating mechanisms through which spiritual leadership exerts influence on employees’ proactive workplace behaviors (e.g., Fry et al., 2005; Fry, 2008; Sanders, 2017), this study also investigates the route through which spiritual leadership influences employees to identify opportunities, show initiative, and/or take action.

Proactive workplace behavior can be defined as a “process whereby individuals recognize potential problems or opportunities in their work environment and self-initiate change to bring about a better future work situation” (Parker and Collins, 2010, p. 636). Employees’ proactive workplace behavior is getting increasingly important for organizations to adapt to a volatile business environment and obtain a competitive advantage (Vough et al., 2017). Drawing on self-determination theory (SDT) (Ryan and Deci, 2000a; Parker and Collins, 2010), our study uncovers the mediating mechanisms through which spiritual leadership influences proactive workplace behavior by suggesting organizational identification and psychological safety as two sequential mediators. In other words, when being inspired by spiritual leaders, employees are more likely to perceive a higher level of organizational identification and psychological safety, which would serve as “reasons” to exhibit proactive behaviors.

**FIGURE 1:**
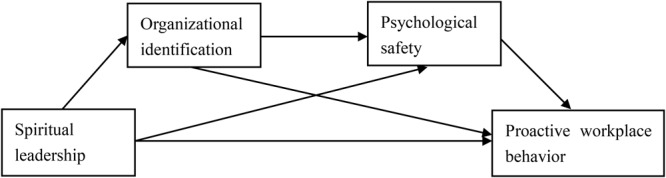
The conceptual model.

In summary, our research should contribute to the fields of spiritual leadership and proactive workplace behavior in the following regards. Firstly, it extends the existing impact of spiritual leadership to proactive workplace behavior and explores how and why spiritual leadership has such a positive effect on employees’ proactive workplace behavior, thereby enriching the literature on spiritual leadership. Moreover, this study contributes to the literature on proactive workplace behavior, enhancing our understanding of antecedents that may facilitate proactive workplace behavior by focusing on workplace spirituality. Secondly, through the application of SDT, this study investigates the impacts of two sequential mediators (organizational identification and psychological safety), thereby shedding lights on the mediating mechanisms through which spiritual leadership exerts influences on proactive workplace behavior. Thirdly, it is unclear whether results from prior studies conducted in the West would hold in the Eastern context. We conduct the study in China, a society with relatively high power distance and collectivist values and where employees are vulnerable to the unequal power held by supervisors and susceptible to influences from organizations (House et al., 2004; Gong et al., 2012). In such a cultural context, perceived psychological safety based on identification relationship is likely to be particularly important. Figure 1 illustrates the overall conceptual model of our study.

## Theory and Hypotheses

### Self-Determination Theory

Scholars (e.g., McGregor, 1960; Herzberg, 1966) have assumed that work can be inherently enjoyable and interesting, rather than simply tiring and miserable, as perceived by employees. This view is represented by and developed in theories of self-determination and intrinsic motivation (e.g., Deci et al., 1975; Deci and Ryan, 1985). SDT is a macro theory of human motivation and personality that concerns people’s inherent growth tendencies and innate psychological needs. It is concerned with the motivation behind choices people make without external influence and interference (Deci and Vansteenkiste, 2004). SDT focuses on the degree to which an individual’s behavior is self-motivated and self-determined.

Motivation involves the forces arousing a person’s interest and enthusiasm in pursuing certain sets of actions or behaviors. Motivation is mainly about energizing an individual’s behavior, the channels that directs such actions or behaviors as well as how such action can be sustained in the long run (Sansone and Harackiewicz, 2000). The basic components of a general motivation process include expectations or needs, behavior, performance or goals, feedback, and rewards (Deci, 1971). Ryan and Deci (2000b) suggest that the reason why individuals initiate or sustain certain actions is that they believe these actions would help them achieve expected outcomes or reach their goals. Motivation in the workplace can result from the desire to receive and achieve individual or group rewards from a favorable environment that the leaders create. These desires cause people to exert significant effort and exhibit cooperative behaviors (Kanfer, 1990).

Key studies which led to the emergence of SDT included research on intrinsic motivation (e.g., Mitchell and Daniels, 2003; Grant, 2008). Intrinsic motivation is “interest and enjoyment of an activity for its own sake and is associated with active engagement in tasks that people find interesting and fun and that, in turn, promote growth and satisfy higher order needs” (Deci, 1971, p. 105). Such higher order needs include the needs for autonomy, competence and relatedness (Deci, 1971), which motivate the self to initiate behavior and specify nutriments that are essential for psychological health and well-being of an individual. In other words, intrinsic motivation requires, to some extent, self-management or autonomy in the workplace, and an intrinsically motivated employee should feel more relatedness and competence by working in an empowered environment that drives the group or organization activities to a meaningful direction and purpose (Giacalone and Jurkiewicz, 2003). Prior studies have suggested a set of variables that are related to intrinsic motivation, including performance, better learning and well-being (Benware and Deci, 1984; Deci and Ryan, 1985; Kruglanski et al., 2018).

### Spiritual Leadership

Spiritual leadership is largely based on an SDT model. The three key elements of spiritual leadership include vision, hope/faith, and altruistic love (Fry and Cohen, 2009). This firstly entails a vision created by spiritual leaders wherein followers feel a sense of calling, i.e., they feel their life is meaningful and they can make a difference. Second, spiritual leadership incorporates altruistic love, which makes both leaders and team members care, concern and appreciate each other. Through altruistic love, team members will feel they are being accepted and understood by their teams, thus developing a sense of membership. Prior research has evidenced that spirituality programs at workplaces contribute to a series of individual outcomes including well-being, improved productivity and commitment and reduced turnover/absenteeism (Fry and Slocum, 2008). In sum, spiritual leaders can be regarded as the role models in terms of communicating the vision and goals, cultivating followers’ faith and hope, and realizing followers’ spiritual needs for their goals and well-being (Fairholm, 1996; Modaff et al., 2008; Crossman, 2010).

### Spiritual Leadership and Proactive Work Behavior

As mentioned previously, unlike traditional leadership which influences people through power, control and even fear, spiritual leaders should motivate followers through values, vision, and altruistic love (Daft and Lengel, 1998; Fry, 2003). From this perspective, spiritual leadership can be regarded as an intrinsically motivating force that makes employees feel energized, alive and connected in the workplace. The employees who are intrinsically motivated by spiritual leaders would then feel that their spiritual needs have been satisfied. As a result, they will generate more feelings of fun, care and attraction for work so that they become more productive and committed (Giacalone and Jurkiewicz, 2003).

In this study, we suggest that spiritual leadership focuses mainly on the observable and behavioral aspects of spirituality and may contribute to followers’ proactive workplace behavior (Reave, 2005). Proactive workplace behavior can be viewed as an autonomous course of action performed and promoted by employees (e.g., Bindl and Parker, 2010; Tornau and Frese, 2013). It involves anticipatory and self-initiated action that aims at changing the situation and/or oneself. Examples of proactive behaviors include taking charge, proactive problem solving and proactive feedback seeking, etc. As noted above, spiritual leadership helps paint and communicate a vision for a bright future, through which followers would believe in this vision and be more likely to engage in initiating changes and contribute to activities that are helpful for organizational transformation (Benefiel, 2005). From this perspective, spiritual leadership can serve as an encouraging force of proactive workplace behavior. Drawing on SDT theory, the intrinsic motivation aroused by spiritual leadership could increase employees’ potential to/interest in identifying potential problems and initiating changes without feeling the burden of fear, worry, or control. In other words, spiritual leadership energizes people, garners commitment, and gives meaning to employees’ work. Thus, by defining the vision’s journey and destination, encouraging faith and hope, and cultivating loving relationships among team members, spiritual leadership can motivate and mobilize employees to engage further in proactive workplace behaviors to bring about a better future for their organizations. In contrast, leaders who ignore the spiritual needs and emotional wellbeing of employees may cause them to feel loneliness, confusion, and a lack of joy and happiness, thereby restraining followers’ intrinsic motivation, appreciation, and enthusiasm for the vision of the organization. As a result, their proactive workplace behavior may suffer (Fry, 2003). Therefore, we propose the following:

Hypothesis 1: Spiritual leadership is positively related to employees’ proactive workplace behavior.

### The Mediating Role of Organizational Identification

Some prior research evidence indicates that leaders’ behavior does not directly influence employees’ behavior; rather, it indirectly does through employees’ cognitive and psychological processes (i.e., Strauss et al., 2009; Cho and Dansereau, 2010). Accordingly, we predict that spiritual leadership may indirectly influence employees’ proactive workplace behavior through other variables, such as a person’s organizational identification, which is defined as “a perceived oneness with an organization and the experience of the organization’s successes and failures as one’s own” (Gioia et al., 2000, p. 65). Accordingly, organizational identification may happen “when an individual’s beliefs about his or her organization become self-defining... [so as to] integrate beliefs about one’s organization into one’s identity” (Pratt, 1998, p. 172).

As it is believed that leadership styles could be a source for organizational identification (Riketta, 2005), we suggest that identification with one’s organization is likely to be aroused through spiritual leadership. According to SDT, spiritual leadership not only intrinsically motivates, empowers, and energizes others, but also fosters a spirit of trust, cooperation, mutual caring and understanding (Fry, 2003). Through such recognition and positive feelings, employees who work with spiritual leaders will feel stronger senses of calling, membership and belongingness, which may help to elicit employees’ sense of organizational identification. In other words, through vision, hope, faith, and altruistic love, spiritual leadership provides a joyful and inclusive work environment, which makes organizational identification more attractive so that employees identify more with their organizations.

Meanwhile, organizational identification should also have a positive influence on employees’ proactive workplace behavior. As an individual’s vision, values, and identification become more integrated with that of their organization, they are more likely to be proactive in the workplace and put forth the maximum effort required to get the job done for the better performance and secure future of their organization.

Building on these theoretical arguments, we reason that spiritual leadership enables followers to experience a sense of membership, appreciation, and acceptance through which they become more attracted to their organizational identification and thus begin to develop higher levels of organizational identification. This, in turn, reinforces employees’ motivation and efforts in engaging in proactive workplace behavior. Therefore, we propose the following:

Hypothesis 2: Organizational identification mediates the relationship between spiritual leadership and proactive workplace behavior.

### The Mediating Role of Psychological Safety

The conditions of psychological safety have been recognized by researchers as an important state or condition at work that may influence an employee’s behavior. Psychological safety is defined as “individuals’ perceptions of the consequences of taking interpersonal risks in their work environment” (Edmondson, 2002, p. 5). It describes the extent to which that “people are comfortable being themselves” and “feel able to show and employ one’s self without fear of negative consequences to self-image, status, or career” (Kahn, 1990, p. 708; Edmondson, 1999, p. 354). Nembhard and Edmondson (2006) suggest that previous studies have found that leadership can contribute to followers’ psychological safety. In particular, Edmondson et al. (2004) contend that employees in the workplace are more likely to develop a higher level of psychological safety when their leaders exhibit availability, openness and accessibility.

In a similar vein, spiritual leadership may also help employees to perceive a higher level of psychological safety. Followers who work with spiritual leaders should be less fearful, more ethical, and more committed. In addition, under spiritual leadership, mutual respect and interpersonal trust are more likely to be promoted throughout the organization. As high-quality interpersonal relationships and trust play an important role in developing a high level of psychological safety (Carmeli and Gittell, 2009), employees’ perception of psychological safety could be developed by spiritual leadership. Spiritual leaders encourage followers to believe that they are appreciated and respected by their organization, enabling them to feel more comfortable with being and expressing themselves or taking risks. In this respect, drawing on SDT, spiritual leadership is important because it emphasizes altruistic love, promotes trust, and fosters employees’ sense of appreciation and acceptance so that followers could be motivated to engage in interpersonal risk-taking and are less likely to experience fear of unfavorable outcomes. This suggests a positive relationship between spiritual leadership and followers’ perception of psychological safety.

Meanwhile, a work environment wherein it is safe to take personal risks and express new ideas is critical for followers’ creativity and proactivity (Wang et al., 2018). Specifically, higher levels of psychological safety facilitate proactive and learning behaviors, such as experimenting and making improvements, in that it alleviates employees to come up with new ideas, identify new problems, and initiate changes without feeling fear of the negative consequences or unexpected outcomes. Employees may feel more comfortable when proposing and initiating changes when they perceive a higher level of psychological safety. Essentially, we predict that this linkage with psychological safety could explain the relationship between spiritual leadership and proactive workplace behavior. Together, these sets of arguments suggest the following hypothesis:

Hypothesis 3: Psychological safety mediates the relationship between spiritual leadership and proactive workplace behavior.

### The Sequential Mediating Roles of Organizational Identification and Psychological Safety

According to SDT, individuals are intrinsically motivated by engaging in activities that they find meaningful and interesting (Deci, 1971; Grant, 2008). In fact, spiritual leadership is a kind of leadership style that is conducive to inspiring positive emotions within the individual, which has been shown to be significantly related to take actions directed toward future impact (Fritz and Sonnentag, 2009). Moreover, by emphasizing vision, faith/hope, and altruistic love, spiritual leadership plays the pivotal role of nurturing higher levels of organizational identification. Such enhanced levels of identification should in turn foster employees’ sense of psychological safety because employees should feel more motivated to take the risk of proposing new ideas or be more willing to take initiatives for the good of the organization that they identify with.

Hence, we contend that employees’ levels of organizational identification are more likely to be promoted through spiritual leadership, which will, in turn, activate increased levels of psychological safety and ultimately lead to enhanced proactive workplace behavior (Hans and Gupta, 2018). In other words, the impact of spiritual leadership on proactive workplace behavior should be transferred via organizational identification and psychological safety. Thus, we suggest the following hypothesis:

Hypothesis 4: Organizational identification and psychological safety sequentially mediate the relationship between spiritual leadership and employees’ proactive workplace behavior.

## Materials and Methods

### Sample and Procedures

A survey was administered to 10 new energy firms in Hubei province, P.R. China. The reason we chose firms in the new energy industry is that these firms are environmentally orientated and require more environmental talents and proactive behavior in the workplace. We first got in touch with the human resource director of each firm and then asked whether their firms are willing to participate in this survey. After getting approval from these firms, we invited team leaders and their subordinates to participate in our survey and guaranteed that their participation was totally voluntary and that their private information was confidential.

Finally, we distributed 230 matching questionnaires to supervisors and employees. At Time 1, employees provided information about their perceived spiritual leadership, organizational identification, and control variables, such as employee gender, age, tenure, and educational background. At Time 2 (3 months later), employees provided information about psychological safety, while their direct supervisors provided information about the proactive workplace behavior of employees. After discarding questionnaires with a response rate of less than two thirds (67%, Chen et al., 2016), we obtained 188 valid questionnaires, reflecting an 81.74% response rate.

Among the participants, there were 97 males, accounting for 51.6% of the total. Most of the employees were aged between 25 and 35 (54.26%), and the job tenure of current employees was less than 3 years (56.91%). More than half of them held a Bachelor’s degree (68.09%).

### Measures

For all measurement instruments, five-point Likert-type scale questionnaires were adopted (from strongly agree = 1 to strongly disagree = 5). Questionnaires were originally constructed in English but administered in Chinese. To ensure the fidelity of the Chinese translation, we adopted Brislin’s (1980) recommendation to use a translation-back translation procedure.

### Spiritual Leadership

This scale was developed by Tang et al. (2014), based on Chinese content. The questionnaire consists of 14 items to assess spiritual leadership from three dimensions: vision, hope/faith, and altruistic love. Example items are: “My department (team) has a statement of the vision of the organization that allows me to perform at my best,” “I trust my organization and I am willing to do whatever it takes to achieve organizational goals,” and “Leaders occasionally communicate with us.” Cronbach’s Alpha of this scale was 0.921.

### Organizational Identification

This scale was developed by Mael and Ashforth (1992). There are six items contained in this scale. Sample items include: “When someone criticizes (my organization), it feels like a personal insult,” and “I am very interested in what others think about (my organization).” Cronbach’s Alpha of this scale was 0.846.

### Psychological Safety

This scale was developed by Edmondson (1999). There are seven items contained in this scale. Some of the items used include: “Members of this team are able to bring up problems and tough issues” and “It is safe to take a risk on this team.” Cronbach’s Alpha of this scale was 0.842.

### Proactive Workplace Behavior

This scale was developed by Parker et al. (2006). This questionnaire consists of eight items to assess proactive idea implementation and proactive problem solving. Example items are: “Implementing ideas for improvements oneself” and “Suggesting ideas for improvements to colleagues.” Cronbach’s Alpha of this scale was 0.883.

### Control Variables

Control variables such as employee gender, age, tenure, and educational background, were collected because these factors were relatively important to employee attitude and behaviors based on previous research (Wang et al., 2018).

## Analysis and Results

### Measurement Model

Using the AMOS20.0 software, confirmatory factor analysis was used to test the goodness of fit of the measurement model. When compared with the one-factor model, two-factor model, and three-factor model, the four-factor model, which consists of spiritual leadership, organizational identification, psychological safety, and proactive workplace behavior, was found to be the best fit. As shown in Table 1, the fitting indexes are significantly better (χ^2^/df = 1.653, RMSEA = 0.059, CFI = 0.907, IFI = 0.908). It indicates that the five constructs proposed in this study are well differentiated and can be followed by hypothesis testing.

**Table 1:** Assessment of alternative measurement models (*N* = 188).

Model types	χ^2^*/df*	Changeχ^2^	RMSEA	CFI	IFI
Four-factor model (expected model)	1.653	–	0.059	0.907	0.908
The best three-factor model (1: SL; 2:OI + PS; 3:PB)	1.695	27.81 (3)	0.061	0.900	0.902
The best two-factor model (1: SL+OI; 2: PS+ PB)	2.102	223.62 (2)	0.077	0.841	0.843
One-factor model (1:SL+OI+PS+PB)	2.192	51.042 (1)	0.080	0.828	0.830

*SL, spiritual leadership; OI, organizational identification; PS, psychological safety; PB, proactive workplace behavior.*

### Descriptive Statistics

The mean, standard deviation, and correlation coefficient of each variable are shown in Table 2. From the correlation coefficient, it can be seen that employee age, tenure and education are significantly positively correlated with proactive workplace behavior (*r* = 0.322, *p* < 0.01; *r* = 0.179, *p* < 0.05; *r* = 0.176, *p* < 0.05, respectively). Additionally, spiritual leadership is significantly positively correlated with organizational identification (*r* = 0.712, *p* < 0.01), psychological safety (*r* = 0.633, *p* < 0.01), and proactive workplace behavior (*r* = 0.650, *p* < 0.01). Furthermore, organizational identification and psychological safety are significantly positively correlated with proactive workplace behavior (*r* = 0.722, *p* < 0.01; *r* = 0.641, *p* < 0.01, respectively). As the data were collected from the same respondents using self-report measures, common method variance (CMV) may have inflated the hypothesized relationships. Thus, we examined the correlation matrix consistent with Bagozzi et al. (1991) that highly correlated variables (*r* > 0.90) suggest evidence of CMV. Table 2 shows no high correlations among these variables.

**Table 2:** Descriptive statistics and Pearson’s correlations among all the variables.

Variables	1	2	3	4	5	6	7	8
1. Employee gender	1							
2. Employee age	-0.019	1						
3. Employee tenure	0.193**	0.624**	1					
4. Employee education	-0.077	0.164*	0.032	1				
5. Spiritual leadership	0.092	0.133	0.071	0.054	1			
6. Organizational identification	0.078	0.216**	0.179*	0.097	0.712**	1		
7. Psychological safety	0.100	0.172*	0.148*	0.119	0.633**	0.713**	1	
8. Proactive workplace behavior	-0.008	0.322**	0.179*	0.176*	0.650**	0.722**	0.641**	1
Mean	1.48	1.90	2.39	2.06	3.79	3.61	3.58	3.79
S.D.	0.50	0.74	1.33	0.67	0.73	0.79	0.74	0.68

*N = 188, ^∗^p < 0.05, ^∗∗^p < 0.01.*

### Hypothesis Testing

To test the hypotheses, we used hierarchical multiple regression first. The results are displayed in Table 3. Specifically, Model 4 suggests that spiritual leadership has a positive effect on proactive workplace behavior (*r* = 0.620, *p* < 0.001), which supports H1. Spiritual leadership has a positive effect on both organizational identification and psychological safety (*r* = 0.695, *p* < 0.001, Model1; *r* = 0.616, *p* < 0.001, Model2, respectively). Moreover, as shown in models 5 and 6, when put separately in the regression model, the mediators of organizational identification and psychological safety have significant positive effects on proactive workplace behavior (*r* = 0.480, *p* < 0.001; *r* = 0.350, *p* < 0.001, respectively). Meanwhile, the coefficient of spiritual leadership decreases when compared with Model 4 (*r* = 0.286, *p* < 0.001; *r* = 0.405, *p* < 0.001, respectively), which suggests partial mediating effects of these two variables. The results support H2 and H3.

**Table 3:** Hierarchical multiple regression analysis.

Variables	Organizational identification	Psychological safety	Proactive workplace behavior			
	**Model1**	**Model2**	**Model3**	**Model4**	**Model5**	**Model6**
**Control Variable**						
Employee gender	0.001 (0.083)	0.035 (0.087)	0.013 (0.098)	–0.054 (0.075)	–0.055 (0.067)	–0.067 (0.070)
Employee age	0.059 (0.072)	0.031 (0.075)	0.318** (0.085)	0.218** (0.065)	0.190** (0.058)	0.208** (0.060)
Employee tenure	0.091 (0.040)	0.075 (0.041)	–0.026 (0.047)	0.006 (0.036)	–0.038 (0.032)	–0.020 (0.034)
Employee education	0.047 (0.061)	0.081 (0.064)	0.125 (0.072)	0.102 (0.055)	0.079 (0.049)	0.074 (0.052)
**Independent Variable**				
Spiritual leadership	0.695*** (0.056)	0.616*** (0.058)		0.620*** (0.050)	0.286*** (0.063)	0.405*** (0.060)
**Mediator**				
Organizational identification					0.480*** (0.059)	
Psychological safety						0.350*** (0.060)
*R*^2^	0.529	0.420	0.120	0.493	0.602	0.564
Change *R*^2^				0.373^∗∗∗^	0.109^∗∗∗^	0.071^∗∗∗^
*F*	40.851^∗∗∗^	26.331^∗∗∗^	6.230^∗∗∗^	35. 397^∗∗∗^	45.555^∗∗∗^	39.026^∗∗∗^

*N = 188, ^∗∗^p < 0.01, ^∗∗∗^p < 0.001. Entries corresponding to the predicting variables are estimations of standard coefficients with standard errors appearing in parentheses.*

Then, in order to test the sequential mediation effects, we used the bootstrapping method (5000 resample) with the 95% confidence interval (CI) to process the data through SPSS 22.0 (Preacher et al., 2010; Hayes, 2013). As shown in Table 4, the total effect of spiritual leadership on proactive work behavior is significant and positive (*r* = 0.613, *p* < 0.001). Besides, the direct effect of spiritual leadership on proactive workplace behavior is significant and positive (*r* = 0.213, *p* < 0.01), which confirms H1. Additionally, the indirect effect of spiritual leadership on proactive workplace behavior is significant and positive (*r* = 0.281, *p* < 0.001; *r* = 0.048, *p* < 0.05; *r* = 0.071, *p* < 0.05), which confirms H2, H3, and H4, respectively. When comparing the three indirect effects of mediators, the first indirect effect (spiritual leadership → organizational identification → proactive workplace behavior) is significantly different with other two indirect effects (*r* = 0.210, *p* < 0.01; *r* = 0.234, *p* < 0.01), while the difference between the second indirect effect and the third indirect effect is not significant.

**Table 4:** Direct and indirect effects and 95% confidence intervals.

Model pathways	Estimated effect	S.E.	BC95% CI lower upper
**Total effects**			
Spiritual leadership → Proactive workplace behavior	0.613^∗∗∗^	0.052	0.509, 0.716
**Direct effects**			
Spiritual leadership → Proactive workplace behavior	0.213^∗∗^	0.067	0.081, 0.344
**Indirect effects**			
Id1: Spiritual leadership → Organizational identification → Proactive workplace behavior	0.281^∗∗∗^	0.064	0.166, 0.422
Id2: Spiritual leadership → Organizational identification → Psychological safety → Proactive workplace behavior	0.071^∗^	0.029	0.024, 0.142
Id3: Spiritual leadership → Psychological safety → Proactive workplace behavior	0.048^∗^	0.022	0.016, 0.106
Id1-Id2	0.210^∗∗^	0.079	0.057, 0.370
Id1-Id3	0.234^∗∗^	0.074	0.093, 0.391
Id2-Id3	0.023	0.024	–0.010, 0.092

*N = 188, ^∗^p < 0.05, ^∗∗^p < 0.01, ^∗∗∗^p < 0.001.*

## Discussion and Implications

As employees nowadays are facing increased levels of pressure and anxiety in the workplace, the question of how to stimulate employees to be more self-orientated and self-motivated toward their job becomes an increasing important issue. Drawing on SDT, this study investigates whether, why and how spiritual leadership led to enhanced proactive workplace behavior. We have overcome common method bias by using two different collection points and added to the growing literature examining spiritual leadership in non-Western settings. The findings suggest that spiritual leadership has a significant positive effect on proactive workplace behavior. Additionally, both organizational identification and psychological safety mediate the relationship between spiritual leadership and proactive workplace behavior. Furthermore, spiritual leadership positively influences organizational identification, and such organizational identification nurtures psychological safety, which in turn fosters employees’ proactive workplace behaviors. The findings of this study proves the positive effect of spiritual leadership on employees’ behaviors in the workplace, which is consistent with prior research findings (e.g., Chen and Yang, 2012; Afsar et al., 2016; Fry et al., 2017). Moreover, this study also reveals organizational identification and psychological safety as two sequential mediators through which spiritual leadership contributes to employees’ proactive behaviors. In other words, by fostering a spirit of trust, cooperation, mutual caring and understanding, spiritual leadership enables employees to become identified with an organization and make them feel they are part of the organization they work in, this increases their ability to offer up their opinions without worrying about the negative consequences of expressing different ideas, thus developing higher levels of psychological safety. Such enhanced psychological safety will then lead employees to become more motivated and to take charge at work, or share more ideas for improving organizational performance and thus develop higher levels of proactivity.

### Theoretical Implications

Firstly, this study tested a conceptual model that uniquely integrates spiritual leadership with proactive workplace behavior. Although prior research has explored the effect of leadership style (e.g., transformational leadership, shared leadership) (Den Hartog and Belschak, 2012; Erkutlu, 2012) on proactive workplace behavior, spiritual leadership has been absent from consideration. In order to respond to the call for nurturing the spirit at work (e.g., Duchon and Plowman, 2005), this study contributes to the spiritual leadership literature by uncovering the positive effect of spiritual leadership on proactive workplace behavior. Moreover, this study enriches the literature on proactivity and enhances our understanding of the antecedents of proactive workplace behavior, especially in developing countries such as China. In the Chinese context, people tend to develop *guanxi* with their organization and obtain spiritual satisfaction from their organization. Leaders play an essential role in the relationship between employees and organizations, and may facilitate proactive behavior more effectively by creating meaningful work for others as well as a sense of community at work.

Secondly, our study enriches the spiritual leadership literature by uncovering the mediating mechanisms through which spiritual leadership exerts influences on proactive workplace behavior. Although abundant research has been conducted on spiritual leadership, the process through which spiritual leadership affects certain behavioral outcomes has not been sufficiently tested. This study examined the impacts of two sequential mediators from the perspective of SDT. The findings thus not only enrich the spiritual leadership literature but respond to the call from previous researchers (Baer and Frese, 2003; Roussin and Webber, 2012) for integrating spiritual leadership with other relevant theoretical frameworks. Furthermore, by identifying organizational identification and psychology safety as two important mental mechanisms through which spiritual leadership exerts effect on employees’ proactive behaviors such as cooperative behaviors and voice behaviors (e.g., Dukerich et al., 2002; Walumbwa and Schaubroeck, 2009), our study is vital for advancing the existing theoretical models regarding the influence of employees’ psychological status on their behaviors.

### Practical Implications

Our research shows that spiritual leadership plays a pivotal role in fostering followers’ proactive workplace behavior. It proves that to encourage proactive workplace behaviors, leadership does matter. Managers are therefore advised to develop a spiritual leadership style in order to motivate their followers to become happy, committed, and productive employees, thereby exhibiting more proactive behavior in the workplace. To achieve this, managers should place more emphasis on vision, hope/faith, and altruistic love when interacting with employees. This not only helps to energize employees and provide followers with a sense of membership and belonging, but also benefits the performance of the entire organization at large.

Second, as our study uncovers the roles of organizational identification and psychological safety in enhancing employees’ motivation to enable them to be proactive at work. Lately, managers should pay more attention to raise employees’ perceived levels of organizational identification and psychological safety. While these psychological conditions and/or cognitive status are hardly observable, it is still suggested that managers make certain efforts, such as fostering a higher level of mutual trust and frequently showing respect, understanding, and appreciation toward their followers to make organizational identity more attractive and make employees feel more comfortable in expressing themselves. All of these actions should be helpful in nurturing a positive mood among employees through feelings of safety, happiness, and satisfaction within the workplace, which could further facilitate their proactivity, productivity, and performance.

### Limitations and Directions for Future Research

Despite these important findings and implications, there are at least three limitations that should be considered. First, this study mainly focused on the underlying mechanism through which spiritual leadership influences proactivity, but ignores some possible contextual factors which may influence the extent to which spiritual leadership influences employees’ proactive workplace behavior. In other words, we still have limited understanding of when spiritual leadership could enhance employee proactive workplace behavior. Future research is therefore suggested to identify some important moderators that may influence the relationship between spiritual leadership and proactivity. Second, although we uncovered the channels (i.e., organizational identification and psychological safety) that direct spiritual leadership’s influence on proactive workplace behavior, these may only partially explain the total magnitude of the focal relationship. Further research could be conducted to consider and test other alternative mediators, such as employee attitudes, emotions, or traits. Third, as this research was conducted in a Chinese context, future research should be conducted in other countries to increase the generalization of the findings.

## Author Contributions

SC and GZ designed the study materials and collected the data. WJ and FC processed the data. SC and WJ drafted the earlier versions of the manuscript. GZ and FC thoroughly commented on these versions including further intellectual content. All authors were concerned with their analysis and interpretation.

## Conflict of Interest Statement

The authors declare that the research was conducted in the absence of any commercial or financial relationships that could be construed as a potential conflict of interest.
