# Corrigendum: Spiritual Leadership on Proactive Workplace Behavior: The Role of Organizational Identification and Psychological Safety

**DOI:** 10.3389/fpsyg.2019.02056

**Published:** 2019-09-10

**Authors:** Silu Chen, Wanxing Jiang, Guanglei Zhang, Fulei Chu

**Affiliations:** ^1^School of Economics and Business Administration, Central China Normal University, Wuhan, China; ^2^School of Business Administration, Shanghai Lixin University of Accounting and Finance, Shanghai, China; ^3^School of Management, Wuhan University of Technology, Wuhan, China; ^4^College of Business Administration, Capital University of Economics and Business, Beijing, China

**Keywords:** spiritual leadership, proactivity, organizational identification, psychological safety, intrinsic motivation, self-determination theory

In the published article, there was an error in affiliation 2. Instead of “School of Business Administration, Shanghai Linxin University of Accounting and Finance, Shanghai,” it should be “School of Business Administration, Shanghai Lixin University of Accounting and Finance, Shanghai, China.”

Additionally, in the original article, there was an error. The software used was incorrectly provided as “AOMS20.0” instead of “AMOS20.0”.

A correction has been made to the section ***Analysis and Results***, subsection ***Measurement Model***:

“Using the AMOS20.0 software, confirmatory factor analysis was used to test the goodness of fit of the measurement model.”

The authors apologize for this error and state that this does not change the scientific conclusions of the article in any way. The original article has been updated.

